# Joint association between body fat and its distribution with all-cause mortality: A data linkage cohort study based on NHANES (1988-2011)

**DOI:** 10.1371/journal.pone.0193368

**Published:** 2018-02-23

**Authors:** Bin Dong, Yang Peng, Zhiqiang Wang, Odewumi Adegbija, Jie Hu, Jun Ma, Ying-Hua Ma

**Affiliations:** 1 School of Public Health, Peking University Health Science Center, Beijing, P. R. China; 2 Centre for Chronic Disease, School of Medicine, The University of Queensland, Herston, Queensland, Australia; 3 Institute for Urban Indigenous Health, Brisbane, Queensland, Australia; Memorial University, CANADA

## Abstract

**Objective:**

Although obesity is recognized as an important risk of mortality, how the amount and distribution of body fat affect mortality risk is unclear. Furthermore, whether fat distribution confers any additional risk of mortality in addition to fat amount is not understood.

**Methods:**

This data linkage cohort study included 16415 participants (8554 females) aged 18 to 89 years from National Health and Nutrition Examination Survey III (1988–1994) and its linked mortality data (31 December 2011). Cox proportional hazard models and parametric survival models were used to estimate the association between body fat percentage (BF%), based on bioelectrical impedance analysis, and waist-hip ratio (WHR) with mortality.

**Results:**

A total of 4999 deaths occurred during 19-year follow-up. A U-shaped association between BF% and mortality was found in both sexes, with the adjusted hazard ratios for other groups between 1.02 (95% confidence interval: 0.89, 1.18) and 2.10 (1.47, 3.01) when BF% groups of 25–30% in males and 30–35% in females were used as references. A non-linear relationship between WHR and mortality was detected in males, with the adjusted hazard ratios among other groups ranging from 1.05 (0.94, 1.18) to 1.52 (1.15, 2.00) compared with the WHR category of 0.95–1.0. However in females, the death risk constantly increased across the WHR spectrum. Joint impact of BF% and WHR suggested males with BF% of 25–30% and WHR of 0.95–1.0 and females with BF% of 30–35% and WHR <0.9 were associated with the lowest mortality risk and longest survival age compared with their counterparts in other categories.

**Conclusions:**

This study supported the use of body fat distribution in addition to fat amount in assessing the risk of all-cause mortality.

## Introduction

Recent studies demonstrated the U-shaped association of body mass index (BMI) and all-cause mortality. These findings suggested both underweight and obesity were related with the increased risk of mortality, and the lowest risk of mortality in the whole population was observed with a BMI of around 25 kg/m^2^ [1–3]. However, BMI cannot discriminate between lean tissue and body fat mass [4]. Some researchers used the skinfold thickness to estimate the body fat[5],but the skinfold thickness is a measure of subcutaneous fat mass and therefore it is not able to measure intra-abdominal depots. It is unclear whether the U-shaped pattern of the relationship between fat and mortality persists, when more direct measurement of body fat percentage (BF%), rather than BMI and skinfold thickness, is used. Furthermore, the optimal cut-off points for the BF% that associated with the lowest mortality risk remain unclear [6, 7].

In addition, BMI cannot determine the patterns of fat distribution within the body which were identified with other indicators, such as waist-hip ratio (WHR) [8]. There is increasing evidence that variation in location of body fat is associated with different risk of cardiovascular disease, diabetes and cancer [9, 10]. Previous studies have also reported the association between WHR and mortality[11].The mortality risk varies among individuals with the same BF% but different body fat distribution has not been fully characterized. The joint association between body fat amount and fat distribution with the risk of mortality requires further exploration.

In this study, the public released data of National Health and Nutrition Examination Survey (NHANES) III from 1988–1994 which were linked to the mortality data until 31 December 2011 were used. We hypothesised that BF% and WHR have a combined impact on premature death risk. These findings may improve our understanding on the association between body fat and premature mortality, and contribute to current inconsistent recommendations regarding optimal BF%.

## Subjects and methods

### Study population

Public released data of NHANES III were used in this cohort study, which were collected by National Center for Health Statistics of U.S. Centers for Disease Control and Prevention. The detail of the survey design was described elsewhere [12]. In brief, NHANES III was a nationally representative cross-sectional survey conducted from 1988 until 1994. This survey included a national, complex multistage, clustered, stratified probability sample of the civilian, noninstitutionalized American population. Data collection occurred during a home interview and a health examination conducted in a mobile examination center. The Institutional Review Board at the Centers for Disease Control and Prevention approved the study design and all participants provided written informed consent [12]. Our project of analysing the survey data was approved by the School of Low Risk Ethical Review Committee of the University of Queensland (2016-SOMILRE-0161).

A total of 30818 adolescents and adults were examined in NHANES III with an overall examined response rate of 78%. Among 19370 adults aged between 18 and 89 years, pregnant women were excluded because they were not eligible for the bioelectrical impedance analysis (BIA) procedure (n = 322). Another 25 individuals without follow-up data were also removed from the sample. Additionally, participants with missing data of either WHR or BIA resistance were excluded as well (n = 2608), which lead to the final sample of 16415 participants for analysis.

### Baseline measurements

During the home interview, self-reported sex, age, ethnicity (non-Hispanic white, non-Hispanic blacks, Mexican American, and the other),year of education, urbanization (metropolitan area, and other), and family income (< $20000/year, and ≥ $20000/year) were recorded. Smoking status, including current, former, and never smoking, was identified according to the use of cigarettes, cigars, or pipe tobacco. Participants who smoked at least 100 cigarettes, 20 cigars, or 20 pipes of tobacco in their lifetime, but no longer smoked in the home interview were considered as former smokers. Leisure-time physical activity, including jogging or running, riding a bicycle or exercise bicycle, swimming, aerobic dancing, other dancing, calisthenics or floor exercises, gardening or yard work, weight lifting, and other activities, and the frequency of doing these activities was recorded. These information were used to calculate the physical activity, which was further classified as 6 groups (0, 1–5, 6–10, 11–20, 21–30, and ≥ 31 times/month). The consumption frequency and unit of alcoholic beverages (beer, wine, liquor) over the past month also were assessed, which was additionally classified into 6 groups (0, > 0 – ≤ 5, > 5 – ≤ 10, > 10 – ≤ 20, > 20 – ≤ 30, and > 30 drinks/month). A participant was identified as hypertensive if he/she had an average seated blood pressure ≥140/90 mm Hg [13], or was told had hypertension/ high blood pressure by a doctor or a health professional.

History of pre-existing chronic diseases, including diabetes, heart attack, heart failure, stroke, chronic bronchitis, emphysema, and cancer, was also collected. Individuals with elevated plasma glucose (≥ 7 mmol/l) or elevated HbA1c (≥ 6.5%), as well as those who were diagnosed by doctors, were identified as diabetic patients [14]. All other chronic conditions were diagnosed by doctors.

Trained technicians measured height to the nearest 0.1cm using a fixed stadiometer, and measured circumference measures to the nearest 0.1 cm using a steel measuring tape. Waist circumference (WC) was measured at the level of iliac crest at the end of a normal expiration. Hip circumference (HC) was measured at the widest circumference around the buttocks. The WHR was calculated as WC (cm) divided by HC (cm).

Body composition was also examined. Participants had a single, tetrapolar BIA measurement of resistance (Res) and reactance at 50 kHz taken between the right wrist and ankle while in a supine position, using Valhalla 1990B Bio-Resistance Body Composition Analyzer (Valhalla Scientific, San Diego, CA, USA) [12]. Because the equations for calculating fat-free mass (FFM) were developed based on RJL bioelectrical impedance analyzers (RJL, Clinton Twp, MI, USA), the Valhalla resistance value for each NHANES III subject was converted to an equivalent RJL resistance value according to the equations developed by WC Chumlea *et al*. as follows [15]:

For males: RJL resistance = 2.5 + 0.98 Val resistance (*r*^2^ = 0.996, root-mean-square error (RMSE) = 5.0 ohms)

For females: RJL resistance = 9.6+ 0.96 Val resistance (*r*^2^ = 0.993, RMSE = 5.3 ohms)

The FFM prediction equations developed based on RJL resistance data were listed below, which were further used to estimate the percentage of body fat (BF% = ((weight—FFM)/ weight) × 100%) [16].

For males: FFM = -10.678 + 0.262 weight + 0.652 height^2^/RJL resistance + 0.015 RJL resistance (*r*^2^ = 0.90, RMSE = 3.9kg)

For females FFM = -9.529 + 0.168 weight + 0.696 height^2^/RJL resistance + 0.016 RJL resistance (*r*^2^ = 0.83, RMSE = 2.9kg)

### Follow-up

Mortality status and data of death were recorded in the NHANES linked National Death Index (NDI) public-access files through December 31, 2011. We merged the baseline data from NHANES III with follow-up data from the National Death Index. For those who died before December 31, 2011, survival time ended at the age of death, otherwise they were censored at the age attained by the end of 2011.

### Statistical analysis

To explore the non-linear association between BF% and WHR with all-cause mortality, adiposity indicators (BF% and WHR) were converted into restricted cubic spline variables with 4 knots. The values of these knots were 5th, 35th, 65th, and 95th percentiles. The partial correlations between BF% and different adiposity indicators were investigated when age and ethnicity were controlled. As previous studies suggested the relationships between BF% and WHR with mortality and other adverse outcomes may differ between sexes [7, 17, 18], the analyses were performed separately for both sexes. Cox proportional hazard models were performed to obtain the hazard ratios and their 95% confidence intervals (CIs) in males and females, after adjustment for baseline age and ethnicity. For the purpose of comparison, both BF% and WHR were classified into 7 categories. Hazard ratios and their 95% CIs were calculated when the corresponding median groups were used as the reference group. We adjusted for baseline age and ethnicity in initial models, and further adjusted for household income, year of education, urbanization, physical activity, alcohol intake, smoking status, and hypertension. These results were presented graphically. To assess the joint influence of BF% and WHR on risk of mortality, participants were categorized into 9 groups based on BF% and WHR to estimate the hazard ratios when the groups with lowest risk of mortality were used as the reference. In addition, the survival ages, as well as their differences, were also evaluated using parametric survival models with the Stata commands of *streg*, when the data were set with the “origin time” of zero and the “enter time” as the age of baseline examination. The Bonferroni method was used when multiple comparisons were conducted. In sensitivity analysis, to limit the effects of reverse causality, we assessed the relationship in non-smokers who had follow-up duration ≥ 5 years and without pre-existing chronic diseases, because these factors can themselves affect adiposity [1]. Statistical relationships were considered significant for *P* values < 0.05. All analyses were performed using Stata 14 (College Station, Texas, USA).

## Results

A total of 16415 individuals were included in this study. During a median follow-up time of 19 years, 2673 and 2326 deaths occurred in males and females, respectively. **Table 1** shows the baseline characteristics by sex. While the mean BF% of females was 12.3% higher than that in males, males had a larger WHR than females.

**Table 1:** Baseline characteristics of participants, NHANESIII.

Variable		Male		Female	
		N	Mean (SD)/n (percentage)	N	Mean (SD)/n (percentage)
Age, year		7861	47.1 (19.5)	8554	47.0 (19.2)
Height, cm		7857	173.5 (7.5)	8550	160.3 (7.1)
Weight, kg		7854	80.1 (16.5)	8537	70.6 (17.7)
BMI, kg/m^2^		7854	26.6 (4.9)	8536	27.4 (6.5)
BIA resistance, ohms		7449	480.7 (66.8)	7935	579.9 (86.2)
Body fat percentage, %		7441	25.5 (6.1)	7917	37.8 (7.4)
Waist circumference, cm		7675	94.8 (13.5)	8314	91.0 (15.4)
Hip circumference, cm		7677	98.8 (9.3)	8322	103.1 (13.0)
WHR		7663	0.96 (0.08)	8308	0.88 (0.08)
Waist-height ratio		7675	0.55 (0.08)	8314	0.57 (0.10)
Year of education, year		7807	10.8 (4.1)	8508	11.0 (3.8)
Ethnicity, % ^§^		7861		8554	
	NH white		3118 (39.7)		3520 (41.2)
	NH black		2145 (27.3)		2477 (29.0)
	Mexican American		2303 (29.3)		2173 (25.4)
	Other		295 (3.8)		384 (4.5)
Household income ≥ $20000/year, % ^§^		7754	4151 (53.5)	8398	4157 (49.5)
Metropolitan area, % ^§^		7861	3924 (49.9)	8554	4167 (48.7)
Physical activity times/month ^§^		7858		8551	
	0		1797 (22.9)		3122 (36.5)
	1–5		1611 (20.5)		1854 (21.7)
	6–10		861 (11.0)		810 (9.5)
	11–20		974 (12.4)		869 (10.2)
	21–30		912 (11.6)		786 (9.2)
	≥ 31		1703 (21.7)		1110 (13.0)
Alcohol intake, drink/month ^§^		7861		8554	
	0		3386 (43.1)		5887 (68.8)
	> 0, ≤ 5		914 (11.6)		1013 (11.8)
	> 5, ≤ 10		608 (7.7)		500 (5.9)
	> 10, ≤ 20		619 (7.9)		402 (4.7)
	> 20, ≤ 30		531 (6.8)		258 (3.0)
	> 30		1803 (22.9)		494 (5.8)
Smoking status, % ^§^		7859		8552	
	Non-smoker		2600 (33.1)		5243 (61.3)
	Former smoker		514 (6.5)		4 (0.05)
	Current smoker		4745 (60.4)		3305 (38.7)
Hypertension, % ^§^		7739	2776 (35.9)	8496	3125 (36.8)
Diabetes, % ^§^		7221	879 (12.2)	7845	1023 (13.0)
Heart attack, % ^§^		7761	455 (5.9)	8437	256 (3.0)
Heart failure, % ^§^		7851	294 (3.7)	8545	261 (3.1)
Stroke, % ^§^		7858	210 (2.7)	8552	205 (2.4)
Chronic bronchitis, % ^§^		7861	320 (4.1)	8553	591 (6.9)
Emphysema, % ^§^		7857	210 (2.7)	8553	110 (1.3)
Cancer, % ^§^		7859	529 (6.7)	8553	619 (7.2)

Note: BIA, bioelectrical impedance analysis; BMI, body mass index; NH, non-Hispanic; SD, standard deviation; WHR, waist-hip ratio.

^§^, values are numbers (percentages, %).

WHR moderately correlated with BF%, and the partial correlation coefficients were 0.54 for males and 0.37 for females when age and ethnicity were controlled for (**Table 2**). Stronger correlations were found between BF% and other adiposity indicators, including BMI, WC, HC and waist-height ratio, with coefficients ranging between 0.64 and 0.85.

**Table 2:** Partial correlation between different adiposity indicators, NHANES III.

Indicators		Body fat percentage, %	WHR	BMI, kg/m^2^	Waist circumference, cm	Hip circumference, cm
Male						
	WHR	0.54 ^‡^				
	BMI, kg/m^2^	0.68 ^‡^	0.57 ^‡^			
	Waist circumference, cm	0.72 ^‡^	0.74 ^‡^	0.93 ^‡^		
	Hip circumference, cm	0.64 ^‡^	0.35 ^‡^	0.90 ^‡^	0.88 ^‡^	
	Waist-height ratio	0.74 ^‡^	0.77 ^‡^	0.92 ^‡^	0.96 ^‡^	0.80 ^‡^
Female						
	WHR	0.37 ^‡^				
	BMI, kg/m^2^	0.85 ^‡^	0.35 ^‡^			
	Waist circumference, cm	0.82 ^‡^	0.63 ^‡^	0.91 ^‡^		
	Hip circumference, cm	0.82 ^‡^	0.16 ^‡^	0.93 ^‡^	0.86 ^‡^	
	Waist-height ratio	0.82 ^‡^	0.63 ^‡^	0.91 ^‡^	0.97 ^‡^	0.81 ^‡^

Note: BMI, body mass index; WHR, waist-hip ratio.

Coefficients were adjusted for age and race.

^‡^, *P*< 0.01.

**Fig 1:**
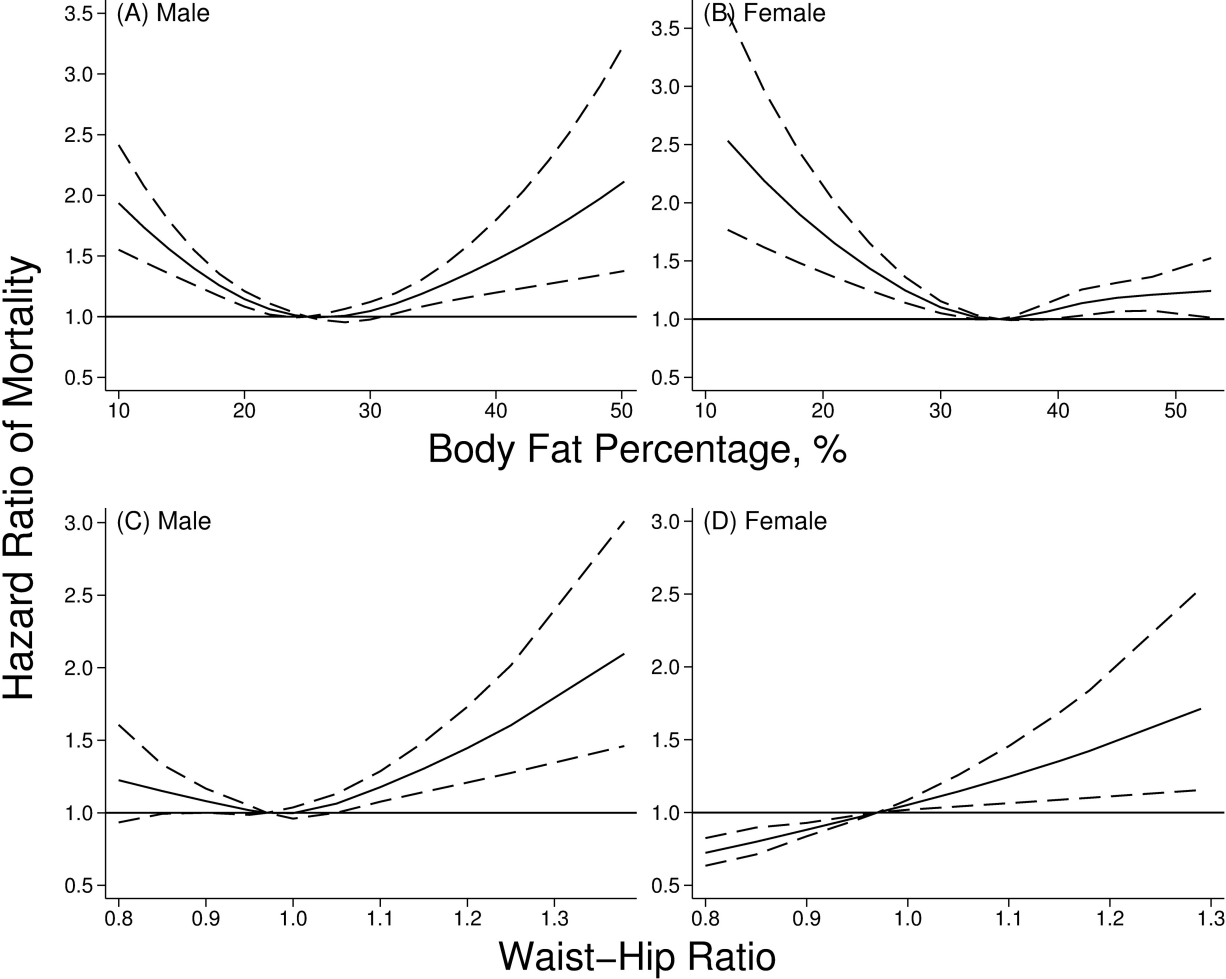
**Association between body fat percentage (A and B) and waist-hip ratio (C and D) with hazard ratio of all-cause mortality in males and females, NHAHES 1988–2011. Notes:** Solid lines and dash lines represent the hazard ratios and their 95% confidence intervals after adjusting for baseline age and ethnicity.

When adiposity indicators were used as continuous variables, a non-linear (U-sharped) relationship between BF% and all-cause mortality was observed in males and females (**Fig 1**) after adjusting for age and ethnicity. The BF% related to the lowest mortality was around 25% in males, and 35% in females. However, the association between WHR and mortality risk differed between males and females when age and ethnicity were adjusted for. While a non-linear association was detected in males with the nadir around 0.97, the mortality risk consistently increased with the increase of WHR in females (**Fig 1**).

**Fig 2:**
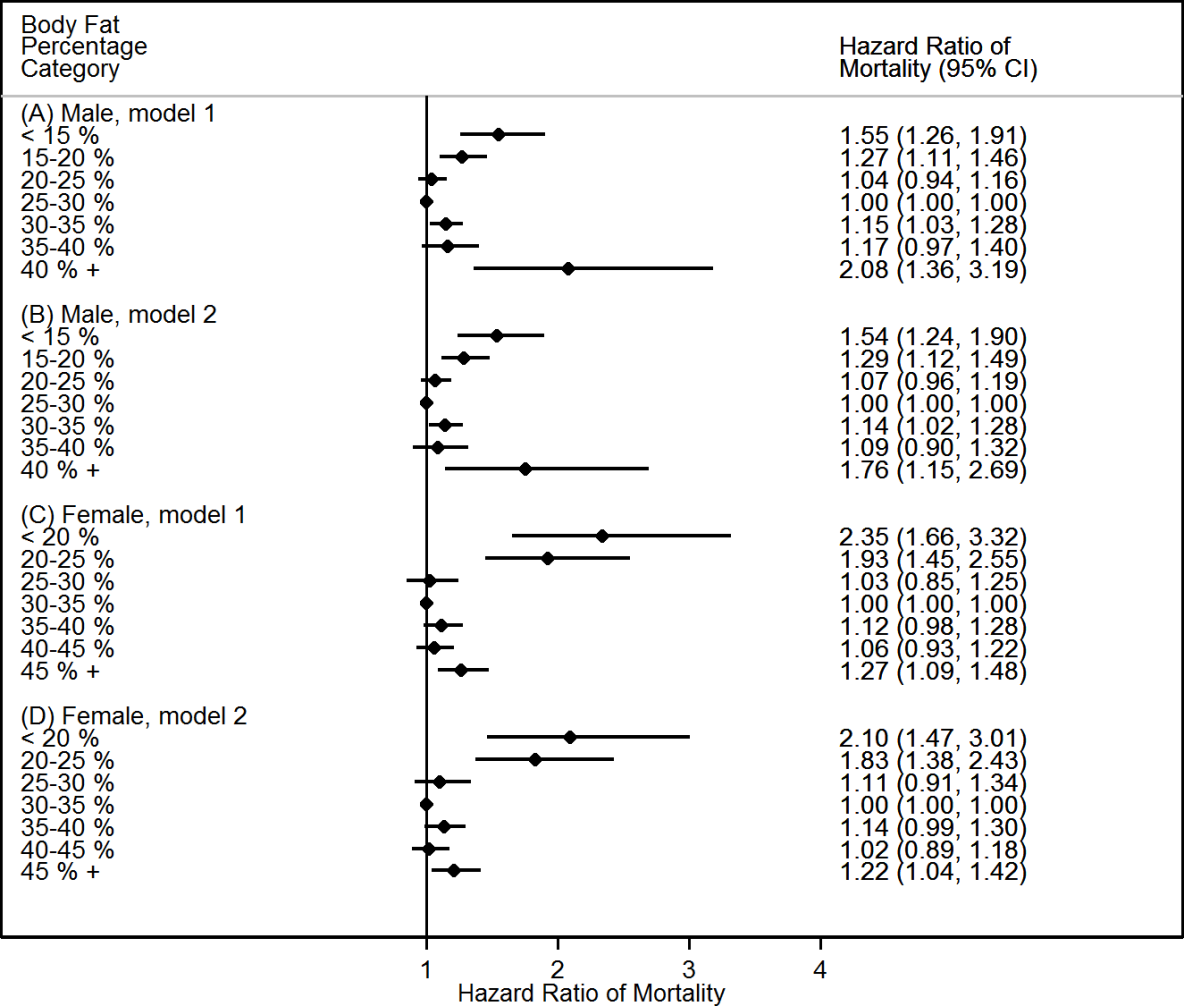
**Hazard ratios of all-cause mortality in males (A and B) and females (C and D) with various body fat percentage categories, NHAHES 1988–2011. Notes:** CI, confidence interval. Body fat percentage categories of 25–30 in males and 30–35 in females were used as the reference groups. Model 1 (A and C) was adjusted for baseline age and ethnicity. Model 2 (B and D) was adjusted for baseline age, ethnicity, household income, year of education, urbanization, physical activity, alcohol intake, smoking status, and hypertension.

**Fig 3:**
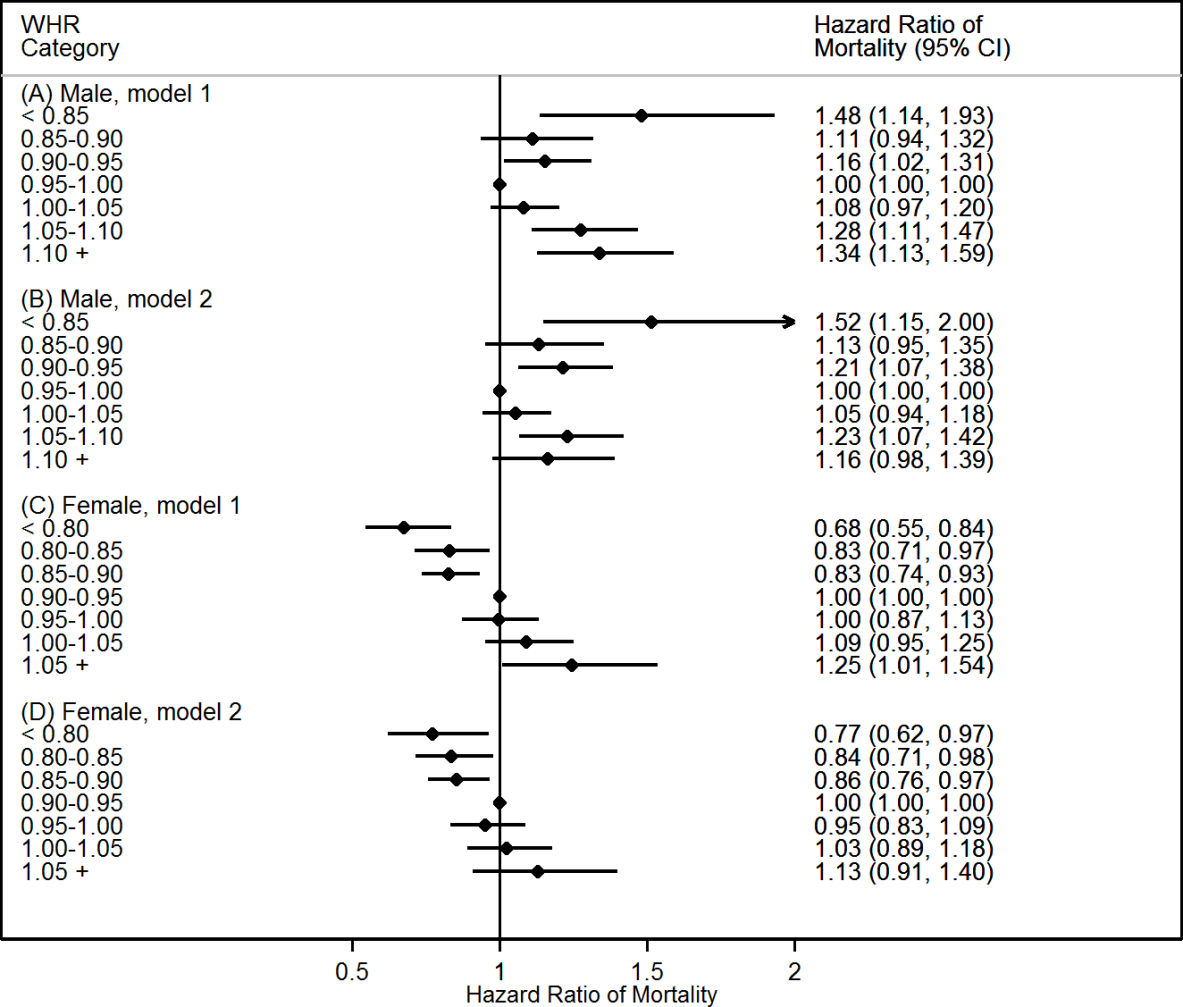
**Hazard ratios of all-cause mortality in males (A and B) and females (C and D) with various waist-hip ratio (WHR) categories, NHAHES 1988–2011. Notes:** CI, confidence interval. WHR category of 0.95–1.00 in males and 0.90–0.95 in females was used as the reference group. Model 1 (A and C) was adjusted for baseline age and ethnicity. Model 2 (B and D) was adjusted for baseline age, ethnicity, household income, year of education, urbanization, physical activity, alcohol intake, smoking status, and hypertension.

Similar patterns between BF% and WHR with mortality were found when the adiposity indicators were used as categorical variables (**Figs 2** and **3**). For instance, risks of mortality in males with BF% <15% increased by 55%, and corresponding risk increased by 108% for BF% >40%, compared with their counterparts with BF% between 25% and 30% (**Fig 2**). Furthermore, those estimates were minimally changed after further adjustment of demographic and lifestyle factors (**Fig 2**). A sensitivity analysis was performed when the participants were limited to non-smokers with follow-up duration ≥ 5 years and without the history of chronic conditions. This restriction led to a large reduction in sample size (more than 70% participants were deleted). Although the estimates declined and failed statistical significance, similar patterns were observed (**Tables A and B in S1 File**).

The joint association between BF% and WHR with mortality and survival age is presented in **Table 3** by sex. After adjustment of the potential confounding factors, with the same BF% category, males within either high or low WHR category were associated with an increased risk of mortality. However, females with small WHR were related to a low risk of mortality. Meanwhile, when the WHR category was given, either high or low BF% was associated with an enhanced mortality risk, which was observed in both sexes. Consistent with the relationships between BF% and WHR with mortality, males within the BF% of 25–30% and WHR of 0.95–1.0 showed the longest survival age compared to their counterparts within other categories, and the difference ranged between 2.2 (95% CI: -2.1, 6.6) and 5.7 (95% CI: 1.8, 9.6) years. Although the differences were not statistically significant, females with BF% of 25–30% and WHR< 0.9 were found to have survived longer than, if not similar with, their peers in other categories.

**Table 3:** Joint association between body fat percentage and WHR with mortality and survival age among American adults, NHAHES 1988–2011.

Body fat percentage category		WHR category	N	Hazard ratio of mortality (95% CI) ^§^	Survival age, year	
					Median survival age (95% CI) ^§^	Difference (95% CI) ^§^
Male						
	< 25%	< 0.95	2201	1.45 (1.20, 1.75) ^‡^	77.0 (75.3, 78.7)	-5.7 (-9.6, -1.8) ^‡^
		0.95–1.0	468	1.15 (0.93, 1.42)	80.5 (78.0, 83.0)	-2.2 (-6.6, 2.1)
		≥ 1.0	458	1.24 (1.02, 1.52) ^†^	79.3 (77.0, 81.5)	-3.4 (-7.6, 0.7)
	25–30%	< 0.95	882	1.19 (0.95, 1.50)	79.8 (77.2, 82.5)	-2.9 (-7.6, 1.8)
		0.95–1.0	536	Reference	82.7 (79.9, 85.6)	Reference
		≥ 1.0	835	1.16 (0.96, 1.39)	80.4 (78.4, 82.5)	-2.3 (-6.1, 1.5)
	≥ 30%	< 0.95	312	1.37 (1.00, 1.88) ^†^	67.3 (73.6, 81.6)	-5.1 (-11.4, 1.2)
		0.95–1.0	361	1.21 (0.95, 1.55)	72.5 (76.7, 82.7)	-3.0 (-8.0, 2.0)
		≥ 1.0	952	1.31 (1.09, 1.58) ^‡^	69.1 (77.0, 80.7)	-3.9 (-7.7, -0.05) ^†^
Female						
	< 30%	< 0.9	1027	1.30 (1.04, 1.63) ^†^	80.0 (77.9, 82.1)	-3.0 (-6.4, 0.5)
		0.9–0.95	68	1.33 (0.94, 1.90)	80.1 (76.4, 83.8)	-2.8 (-8.2, 2.5)
		≥ 0.95	31	1.75 (1.12, 2.72) ^†^	77.1 (72.7, 81.6)	-5.8 (-12.4, 0.7)
	30–35%	< 0.9	1041	Reference	83.0 (80.7, 85.3)	Reference
		0.9–0.95	167	1.03 (0.77, 1.38)	82.9 (79.9, 86.0)	-0.03 (-4.5, 4.4)
		≥ 0.95	142	1.14 (0.87, 1.49)	81.4 (78.6, 84.1)	-1.6 (-5.7, 2.5)
	≥ 35%	< 0.9	2571	1.01 (0.84, 1.22)	83.3 (81.5, 85.0)	0.3 (-2.6, 3.2)
		0.9–0.95	1145	1.29 (1.07, 1.56) ^‡^	80.6 (78.9, 82.3)	-2.3 (-5.3, 0.6)
		≥ 0.95	1281	1.27 (1.06, 1.53) ^†^	80.8 (79.2, 82.4)	-2.2 (-5.0, 0.7)

Note: CI, confidence interval; WHR, waist-hip ratio.

^§^, adjusted for baseline age, ethnicity, household income, year of education, urbanization, physical activity, alcohol intake, smoking status, and hypertension.

^†^, *P*< 0.05

^‡^, *P*< 0.01.

## Discussion

This cohort study of 16415 American adults with 280584 person-years provides evidence that both amount and distribution of body fat are associated with the risk of mortality. While a U-shaped association between BF% and mortality was identified, sex-specific relationships between WHR and mortality were also observed. Furthermore, patterns of these associations persist even when BF% and WHR were combined to predict the risk of mortality. Specifically, males with BF% of 25–30% and WHR of 0.95–1 and females with BF% between 30% and 35% and WHR < 0.9 had the lowest risk of mortality and the longest life expectancy. These findings suggested that current recommendations regarding obesity assessment and management may need to be improved in this population.

Since excess adiposity enhances the risk of adverse conditions, such as cardiovascular diseases and cancer [19, 20], one may expect increased BMI to be related with a high risk of death. In addition, compared with normal weight, studies have also demonstrated that low BMI is associated with chronic pulmonary disease and other respiratory diseases, and result in an increased mortality risk [1–3]. However in most of the previous studies, indirect indicators, such as BMI and skinfold thickness, were used to estimate overall adiposity, and more direct measures, including BIA and dual-energy x-ray absorptiometry (DXA), were rarely applied [5]. Utilization of indirect adiposity indicators is subject to reduced ability to differentiate levels of fatness and leanness among individuals [16]. Zong and colleagues assessed the association between overall body fat measured by dual-energy X-ray absorptiometry (DXA) with all-cause mortality [18]. In their cohort of 9471 American adults with a follow-up duration of 8.8 years, participants in the second BF% quartile had the lowest risk of death compared with their counterparts in either higher or lower quantile [18]. However, that study treated BF% as a categorical variable, and gender disparities in those associations were not reported. In our study, we illustrated the mortality risk across the whole BF% spectrum, and found BF% associated with the lowest risk of mortality, around 25% in males and 35% in females. Additionally, this association changed slightly when life-style behaviours and demographic factors were adjusted for.

Both overall and abdominal adiposity are recommended by clinical guidelines for assessing obesity, and WC is suggested to be used as the indicator of abdominal adiposity [21]. However, the strong correlation between WC and BF% implies that WC is not only an indicator of abdominal adiposity, but also for overall obesity. A number of studies have also observed that WC largely represents variability in intra-abdominal and subcutaneous fat, while variability in HC represents differences in subcutaneous fat, gluteal muscle, and pelvic width [22, 23]. As the consequence, the difference in WHR could be used to distinguish the fat distribution [8]. In the current study, a weaker correlation was found between BF% and WHR, compared with correlations for other adiposity indicators, which suggested WHR may provide additional information regarding body fat distribution. Czernichow *et al*. conducted a meta-analysis and observed the risk of mortality increased across the WHR spectrum [9]. However, that study did not present the association by sex. Using cohort datasets conducted in a Chinese population, a study reported that the risk of mortality increased across the WHR spectrum in women, while the difference in men’s mortality risk was not significant until WHR ≥ 0.95 [17]. Those findings were consistent with our results which showed the sex distinction in WHR related death risk. Although underlying mechanisms remain to be elucidated, research has demonstrated that large HC is associated with the adverse cardio-metabolic profile when WC was controlled for, and that association was stronger among women compared with men [23]. However, the relationship between BF%, as well as WHR, and mortality was largely attenuated in the sensitivity analysis, inferring these relationships may partly be explained by pre-existing conditions. As the sample size significantly declined in the sensitivity analysis, further study with large sample is needed to clarify it.

Extending to above findings, this study found that BF% and WHR were independently associated with mortality, and their joint impact on mortality risk showed males with BF% of 25–30% and WHR of 0.95–1 and females with BF% of 30–35% and small WHR had the longest survival time. These observations were in line with previous studies which showed WHR was associated with mortality independent of BMI [8, 17, 22]. Our findings contribute to the current inconsistent recommendations for assessing adiposity. For instance, while National Institutes of Health classified subjects as obese when BF% exceeds 25% in men and 30% in women [7], World Health Organization expert committee stated “there is no agreement about cut-off points for the percentage of body fat that constitutes obesity” [6]. Other recommendations suggested that it would be beneficial to keep “as lean as possible within the normal range” [24]. The current results advise that the lowest mortality is observed with the BF% around 25% in males and 35% in females, though the possibility that the increased risk among individuals with low BF% is non-causal cannot be ruled out entirely. In addition to stay within an optimal BF%, our results also demonstrated that it is critical to maintain an appropriate body shape which is sex-specific. In current analysis, the differences in expected survival time could be up to 10.8 years among people within same BF% category but different WHR, suggesting WHR could be used to identify people with high risk of mortality in addition to BF% and guide the interventions to reduce the risk of death.

The current study has limitations. Although this study used a large nationally representative longitudinal sample with long duration of follow-up, it is difficult to tease out the confounding effect, such as smoking, in this study. A sensitivity analysis excluding smokers and participants with pre-existing conditions were conducted and the results were similar with current study. However, the small sample size compromise the statistical power and most of those differences were not statistically significant. A study with a larger sample size is desired. In addition, since BF% and WHR were only measured at baseline, the relationship between one measurement on future mortality should be interpreted with caution as it may be impacted by other events and interventions. In addition, we cannot address the impact of any changes in %BF and WHR on the risk of mortality. Finally as this study was conducted on adults living in the United States, studies conducted in other populations are needed to clarify the generalization of these findings.

## Conclusion

Both the amount of body fat and its distribution have been shown to be associated with the risk of disease and mortality. Research on the joint association between body fat amount and fat distribution with mortality based on direct measurement of body fat helps to further elucidate this relationship. Sex-specific optimal levels of BF% and WHR for the lowest risk of mortality in general American adults were also provided. Future study is warranted to confirm these relationships among different populations.

## Supporting information

S1 File **Table A. [Association between body fat percentage and mortality in non-smokers who had follow-up duration ≥ 5 years and without pre-existing chronic diseases, NHAHES 1988–2011.** Note: CI, confidence interval. ^§^, adjusted for baseline age, ethnicity, household income, year of education, urban area, physical activity, alcohol intake, and hypertension. ^†^, *P*< 0.05; ^‡^, *P*< 0.01.] **Table B. [Association between WHR and mortality in non-smokers who had follow-up duration ≥ 5 years and without pre-existing chronic diseases, NHAHES 1988–2011.** Note: CI, confidence interval; WHR, waist-hip ratio. ^§^, adjusted for baseline age, ethnicity, household income, year of education, urban area, physical activity, alcohol intake, and hypertension.]
(DOCX)
