# Supplementary material for: Joint association between body fat and its distribution with all-cause mortality: A data linkage cohort study based on NHANES (1988-2011)
Source: PLoS One. 2018 Feb 23;13(2):e0193368. doi: 10.1371/journal.pone.0193368 (PMC5825095; doi:10.1371/journal.pone.0193368)
Supplement: S1 File — Table A. [Association between body fat percentage and mortality in non-smokers who had follow-up duration ≥ 5 years and without pre-existing chronic diseases, NHAHES 1988–2011. Note: CI, confidence interval. §, adjusted for baseline age, ethnicity, household income, year of education, urban area, physical activity, alcohol intake, and hypertension. †, P< 0.05; ‡, P< 0.01.] Table B. [Association between WHR and mortality in non-smokers who had follow-up duration ≥ 5 years and without pre-existing chronic diseases, NHAHES 1988–2011. Note: CI, confidence interval; WHR, waist-hip ratio. §, adjusted for baseline age, ethnicity, household income, year of education, urban area, physical activity, alcohol intake, and hypertension.] (DOCX) [file pone.0193368.s001.docx]

**Supporting Information**

Joint association between body fat and its distribution with all-cause mortality:

a data linkage cohort study based on NHANES (1988-2011)

Bin Dong^1^, Yang Peng^2^, Zhiqiang Wang^2^,Odewumi Adegbija^2^,

Jie Hu^3^, Jun Ma^1^, Ying-Hua Ma^1*^

^1^ School of Public Health, Peking University Health Science Center, Beijing, P. R. China

^2^ Centre for Chronic Disease, School of Medicine, The University of Queensland, Herston, Queensland, Australia

^3^ Institute for Urban Indigenous Health, Brisbane, Queensland, Australia

***** Corresponding author: yinghuama.bjmu@gmail.com (Y-H M)

**Table A. Association between body fat percentage and mortality in non-smokers who had follow-up duration ≥ 5 years and without pre-existing chronic diseases, NHAHES 1988-2011**

| Body fat percentage category | | N | Hazard ratio of mortality (95% CI) ^§^ | *P* value |
| --- | --- | --- | --- | --- |
| Male | |  |  |  |
|  | < 15 % | 80 | 1.22 (0.61, 2.44) | 0.580 |
|  | 15 - 20 % | 217 | 0.84 (0.46, 1.54) | 0.579 |
|  | 20 - 25 % | 475 | 0.94 (0.65, 1.37) | 0.755 |
|  | 25 - 30 % | 532 | 1 (Reference) |  |
|  | 30 - 35 % | 289 | 0.94 (0.62, 1.40) | 0.745 |
|  | 35 - 40 % | 71 | 1.21 (0.70, 2.11) | 0.493 |
|  | ≥ 40 % | 10 | 3.73 (1.41, 9.88) ^‡^ | 0.008 |
| Female | |  |  |  |
|  | < 20 % | 27 | 2.48 (1.07, 5.77) ^†^ | 0.035 |
|  | 20 - 25 % | 102 | 1.22 (0.58, 2.53) | 0.600 |
|  | 25 - 30 % | 329 | 1.02 (0.68, 1.53) | 0.923 |
|  | 30 - 35 % | 584 | 1 (Reference) |  |
|  | 35 - 40 % | 826 | 1.09 (0.83, 1.45) | 0.527 |
|  | 40 - 45 % | 739 | 0.97 (0.72, 1.29) | 0.815 |
|  | ≥ 45 % | 503 | 1.37 (1.00, 1.89) | 0.052 |

Note: CI, confidence interval.

^§^, adjusted for baseline age, ethnicity, household income, year of education, urban area, physical activity, alcohol intake, and hypertension.

^†^, *P*< 0.05; ^‡^, *P*< 0.01.

**Table B. Association between WHR and mortality in non-smokers who had follow-up duration ≥ 5 years and without pre-existing chronic diseases, NHAHES 1988-2011**

| WHR category | | N | Hazard ratio of mortality (95% CI) ^§^ | *P* value |
| --- | --- | --- | --- | --- |
| Male | |  |  |  |
|  | < 0.85 | 204 | 1.15 (0.49, 2.72) | 0.752 |
|  | 0.85 - 0.9 | 425 | 1.06 (0.58, 1.96) | 0.845 |
|  | 0.9 - 0.95 | 447 | 1.27 (0.81, 1.98) | 0.293 |
|  | 0.95 - 1 | 281 | 1 (Reference) |  |
|  | 1 - 1.05 | 246 | 1.28 (0.85, 1.94) | 0.238 |
|  | ≥ 1.05 | 88 | 1.38 (0.87, 2.20) | 0.172 |
| Female | |  |  |  |
|  | < 0.8 | 631 | 0.82 (0.55, 1.24) | 0.350 |
|  | 0.8 - 0.85 | 732 | 0.97 (0.71, 1.31) | 0.832 |
|  | 0.85 - 0.9 | 863 | 0.95 (0.74, 1.24) | 0.724 |
|  | 0.9 - 0.95 | 547 | 1 (Reference) |  |
|  | 0.95 - 1 | 284 | 0.75 (0.55, 1.01) | 0.056 |
|  | ≥ 1 | 177 | 1.00 (0.75, 1.34) | 0.998 |

Note: CI, confidence interval; WHR, waist-hip ratio.

^§^, adjusted for baseline age, ethnicity, household income, year of education, urban area, physical activity, alcohol intake, and hypertension.
